# An OsKala3, R2R3 MYB TF, Is a Common Key Player for Black Rice Pericarp as Main Partner of an OsKala4, bHLH TF

**DOI:** 10.3389/fpls.2021.765049

**Published:** 2021-10-29

**Authors:** Da-Hye Kim, JuHee Yang, Sun-Hwa Ha, Jae Kwang Kim, Jong-Yeol Lee, Sun-Hyung Lim

**Affiliations:** ^1^Division of Horticultural Biotechnology, School of Biotechnology, Hankyong National University, Anseong, South Korea; ^2^National Academy of Agricultural Science, Rural Development Administration, Jeonju, South Korea; ^3^Department of Genetic Engineering, Graduate School of Biotechnology, Kyung Hee University, Yongin, South Korea; ^4^Division of Life Sciences, Bio-Resource and Environmental Center, Incheon National University, Incheon, South Korea

**Keywords:** anthocyanin, OsKala3, OsKala4, regulation system, rice, tandem repeats in promoters

## Abstract

Rice (*Oryza sativa*) pericarp exhibits various colors due to the accumulation of anthocyanins and/or proanthocyanidins. Previous work revealed that the two basic helix-loop-helix (bHLH) transcription factors OsKala4 and OsRc are key regulators for the black and red pericarp traits, respectively, and their inactivation results in rice with white pericarp. However, their pericarp-specific R2R3 MYB partner remained unknown. Here, we characterized the role of the R2R3 MYB gene *OsKala3* in rice pericarp pigmentation through genetic and molecular approaches. A rice protoplast transfection assay showed that OsKala3 is a nuclear-localized protein. Furthermore, OsKala3 physically interacted with OsKala4 in a yeast two-hybrid analysis. Co-transfection assays in rice protoplasts revealed that OsKala3 and OsKala4 mediate the activation of anthocyanin biosynthetic genes. Notably, the *OsKala3* promoter region exhibited an insertion polymorphism specifically in rice cultivars with black pericarp, creating two tandem repeats while red and white varieties harbor only one. The number of repeats within the *OsKala3* promoter correlated with increased transactivation by OsKala3, thus providing a rationale for the black pericarp characteristic of cultivars with two repeats. These results thus provide evidence for the molecular basis of anthocyanin biosynthesis in rice pericarp and may facilitate the introduction of this beneficial trait to other rice cultivars through marker-assisted breeding.

## Introduction

Anthocyanins and proanthocyanidins (PAs, also referred to as condensed tannins) belong to the flavonoid category of metabolites, one of the largest groups of plant secondary metabolites, and are abundant in the seed coat, leaves, fruits, flowers, and bark of many plant species (Saigo et al., 2020). They have multiple functions, including attracting pollinators or seed dispersal agents, protecting plants against damage from UV radiation and taking part in responses to cold and drought stresses (Harborne and Williams, 2000; Nakabayashi et al., 2014). Both classes of compounds have attracted widespread interest due to their health benefits in preventing chronic human disorders, certain cancers, and cardiovascular diseases (Ross and Kasum, 2002; Zhang et al., 2014; Vinayagam and Xu, 2015).

The anthocyanin biosynthetic pathway has been well characterized, and genes encoding proteins in this pathway have been isolated from many plants (Winkel-Shirley, 2001). Transcriptional regulation of these biosynthetic genes is achieved by a complex comprising MYB-type transcription factors (TFs) of SG5 and SG6, basic helix-loop-helix (bHLH) TFs of the IIIf subgroup and WD-repeat proteins, and the MYB-bHLH-WD40 or MBW complex (Hichri et al., 2011; Petroni and Tonelli, 2011). The MYB TF of this complex contains two conserved imperfect repeats (R2 and R3) in its N terminus, while its C terminus and variable region are responsible for regulatory activity (Nesi et al., 2001; Liu et al., 2016). The bHLH TF is crucial for the formation of transcriptional complexes at the promoters of anthocyanin biosynthetic genes through interaction with the R3 region of its R2R3 MYB partner (Xu et al., 2015; Lim et al., 2017). Finally, the WD40 protein acts as a scaffold protein onto which the complex is assembled and stabilizes the interaction between the MYB and bHLH TFs, rather than having a direct regulatory function (Ramsay and Glover, 2005; Hichri et al., 2011). Several studies have shown that WD40 proteins such as the flavonoid biosynthesis regulator TRANSPARENT TESTA GLABRA1 (TTG1) have pleiotropic effects on additional processes, including the formation of trichomes and root hairs and the production of seed mucilage, and are accordingly expressed in tissues that may either accumulate or lack flavonoids (Zhang et al., 2003; Ramsay and Glover, 2005). As with WD40 proteins, bHLH TFs can regulate, sometimes in a partially overlapping manner, one or more branches of the flavonoid biosynthetic pathway, as well as additional processes such as epidermal cell fate determination. Moreover, the anthocyanin biosynthetic pathway is regulated by a hierarchy of bHLH TFs, such that an upstream bHLH induces the expression of a downstream *bHLH* gene encoding the component of the MBW complex (Petroni and Tonelli, 2011; Montefiori et al., 2015). In contrast, MYB TFs function more narrowly than their WD40 and bHLH TF partners, controlling the specificity of MBW complexes for their cognate target genes to direct the differential accumulation of flavonoids across plant organs and tissues depending on developmental stage and environmental conditions (Gonzalez et al., 2008; Kiferle et al., 2015). Thus, the expression patterns of individual *MYB* genes define the spatial and temporal regulation of the flavonoid biosynthetic pathway, thereby requiring a specific combination of TFs for each tissue and developmental stage.

Wild rice (*Oryza rufipogon*) is a close relative of cultivated rice (*Oryza sativa*) that accumulates anthocyanins in various tissues and PAs in pericarp. Notably, these flavonoid pigments are missing in most cultivated rice varieties, possibly as a consequence of artificial selection for whiter grains. Several rice genes implicated in anthocyanin biosynthesis have been reported (Hu et al., 1996, 2000; Reddy et al., 1998; Sakamoto et al., 2001; Saitoh et al., 2004; Sun et al., 2018; Zheng et al., 2019; Mbanjo et al., 2020; Mackon et al., 2021; Xia et al., 2021). For example, seven putative regulators were isolated and characterized for their contribution to the spatial and temporal regulation of anthocyanin biosynthesis, including the R2R3-MYB gene *OsC1* and the six bHLH genes *OsB1*, *OsB2* (also called *OsKala4*), *OsRa*, *OsRb, OsPa*, and *OsPs*. Genetic analysis demonstrated that *OsC1* is critical for anthocyanin biosynthesis in leaf sheath, apiculus, stigma and hull (Fan et al., 2008; Chin et al., 2016; Sun et al., 2018; Zheng et al., 2019; Meng et al., 2021) and may have been the target of artificial selection for loss of pigmentation during rice domestication, resulting in green leaf sheaths in most cultivated rice plants (Huang et al., 2010). By contrast, *OsKala4* determines anthocyanin biosynthesis in pericarp (Oikawa et al., 2015). A rearrangement in the *OsKala4* promoter region has led to its ectopic expression in the pericarp and the sequential transcriptional activation of anthocyanin biosynthetic genes in this tissue. In addition, the combinatorial activity of OsC1 and OsKala4 promotes anthocyanin biosynthesis in various organs such as leaf sheath, apiculus, and hull, but not in pericarp (Sun et al., 2018). However, ectopic expression of *OsKala4* results in reddish-brown pericarp in the absence of OsC1, indicating that OsKala4 regulates anthocyanin accumulation in this tissue in concert with other MYB-like TFs other than OsC1 (Maeda et al., 2014). Previous studies revealed that introgression of alleles of three genes, Os*Kala1*, Os*Kala3* (also called *OsMYB3*), and Os*Kala4*, from black pericarp cv “Hong Xie Nuo” into cv. “Koshihikari” results in complete conversion of white pericarps into black pericarps. Genetic and molecular analyses determined that Os*Kala1* and Os*Kala4* encode a dihydroflavonol reductase (DFR) and a bHLH TF, respectively (Maeda et al., 2014; Oikawa et al., 2015). However, although *OsKala3* is thought to encode the MYB TF in the MBW complex (Maeda et al., 2014), the underlying molecular mechanism through which it contributes to black color in rice remains unknown.

Here, we identified and characterized the function of *OsKala3* through expression analysis, localization of the encoded protein, and promoter activation assays. Based on *OsKala3* variants between various rice cultivars, we developed a molecular marker for marker-assisted selection and prediction of lines with black pericarp. This study thus helps unravel the regulatory mechanisms underlying anthocyanin synthesis in rice pericarp.

## Materials and Methods

### Plant Materials

Rice seeds were obtained from the Agricultural Genetic Resources Center at the National Institute of Agricultural Science and from the division of Crop Breeding at the National Institute of Crop Science (Jeonju, South Korea). The following four rice cultivars were used for quantitative analysis of gene expression and to identify variation at the *OsKala3* and *OsKala4* loci (categorized according to their pericarp color); white, “Ilmi” (IM); black, “Heugjinju” (HJJ) and “Heugnam” (HN); and red, “Jeogjinju” (JJJ). In addition, 40 rice cultivars (13 white, 16 black, and 11 red) were used to determine the distribution of sequence variation at *OsKala3* and *OsKala4*. These cultivars were grown in the field at the rice experimental station of the National Institute of Agricultural Science and harvested for confirming their pericarp color by dehulling. To isolate rice protoplasts for subcellular localization and transactivation assays, seeds were sown on half-strength Murashige and Skoog (MS) medium and initially kept in the dark for 8–10 days to induce long stems before transfer to long-day conditions (16 h light and 8 h dark) for 1–2 days at 28°C.

### Measurement of Total Anthocyanin Contents

Total anthocyanin contents were determined with various developmental rice seeds stages and vegetative tissues in the seedling stage, according to the method described by Lim et al. (2020). Briefly, samples were ground to powder in liquid nitrogen. Aliquots of 100 mg fresh weight were then mixed in 600 μL extraction buffer (methanol containing 1% [v/v] HCl) for 6 h at 4°C with moderate agitation. An addition of 200 μL water and 200 μL chloroform was followed by centrifugation to pellet plant debris at 14,000 *g* for 5 min at 4°C. After centrifugation, absorbance of the supernatant was recorded at 530 nm (A_530_) and 657 nm (A_657_) using a microplate reader. Anthocyanin content was determined according to the formula *A*_530_ – (0.33 × *A*_657_). Each sample was extracted and measured from three independent experiments.

### RNA Extraction and RT-qPCR

Total RNA was extracted from developing rice seeds at various developmental stages and vegetative tissues at the seedling stages using the Fruit-mate for RNA Purification solution (Takara, Otsu, Japan) and Plant RNA Purification Reagent (Invitrogen, Carlsbad, CA, United States) as described previously (Kim et al., 2018) and then purified using the FavorPrep^TM^ Plant Total RNA Mini Kit (Favorgen, Changzhi, Taiwan). First-strand cDNAs were synthesized from 2 μg of total RNA using amfiRivert cDNA Synthesis Platinum Master Mix (GenDEPOT, Barker, TX, United States). qPCRs were performed using AccuPower 2 × Greenstar qPCR Master Mix (Bioneer, Daejeon, South Korea) and a Bio-Rad CFX96 Detection System (Bio-Rad Laboratories, Hercules, CA, United States), according to the manufacturer’s instructions. The expression levels of all target genes were normalized to that of *Ubiquitin* (*OsUBI*) as an internal reference. Gene-specific primers used for qPCR analysis are listed in Supplementary Table 1. Three independent biological replicates and three technical replicates were performed for each sample.

### Subcellular Localization Analysis

For subcellular localization analysis, the open reading frames (ORFs) of *OsKala3* (BAA23339), *OsKala4* (AB021080), and *OsTTG1* (KAB8088430) from rice cv. HN were PCR-amplified with PrimeSTAR^®^ HS DNA Polymerase (Takara) using gene-specific primer sets (p326-OsKala3-F/R, p326-OsKala4-F/R, and p326-OsTTG1-F/R) and cloned into the p326-sGFP plasmid linearized by *Xba*I digestion using the In-Fusion HD Cloning Kit (Takara). The resulting plasmids encoding C-terminal GFP fusion constructs were sequenced for confirmation of error-free PCR amplification. The plasmids were then introduced into rice protoplasts prepared from rice leaves using a polyethylene glycol (PEG)-mediated transformation procedure, as described by Kim et al. (2018). The accumulation of the fusion proteins (OsKala3-GFP, OsKala4-GFP, and OsTTG1-GFP) was determined 16–20 h after transfection, and images were captured by confocal laser scanning microscopy (Leica TCS SP8; Leica Microsystems, Wetzlar, Germany).

### Transactivation and Yeast Two-Hybrid (Y2H) Assays

Full-length or fragments of the *OsKala3* ORF were PCR-amplified using specific primer sets (Supplementary Table 1) and cloned into pGBKT7 vector, harboring the GAL4 DNA-binding domain (BD), opened at the *Eco*RI restriction site using an In-Fusion HD Cloning Kit (Takara). To detect autoactivation, individual constructs were transformed into yeast strain AH109, following the manufacturer’s instructions (Takara). Transformed yeast cells were grown on synthetic defined (SD) medium lacking Trp and were replica-plated on SD medium lacking Trp, His, and Ade and containing X-α-gal for color development.

To examine the interaction between OsKala3, OsKala4, and OsTTG1, their full-length ORFs were individually cloned into vectors pGBKT7 and pGADT7, harboring the GAL4 DNA-activation-domain (AD), according to the manufacturer’s instructions. Constructs were introduced in pairs into yeast strain MaV203. Colonies were selected on SD medium lacking Trp and Leu and were replica-plated on SD medium lacking Trp, Leu, and His and containing 10 mM 3-amino-1,2,4-triazole (3-AT), a competitive inhibitor of HIS3.

To identify the interaction between OsKala3 and OsKala4, three partial fragments of OsKala3 were cloned into pGBKT7, and the full and partial fragments of OsKala4 ORF were cloned into pGADT7. The resulting constructs were introduced in pairs into yeast strain MaV203. For interaction assays between OsKala4 and OsTTG1, full-length OsTTG1 ORF was cloned into pGBKT7 and introduced with the pGADT7-OsKala4 constructs above in pairs into yeast strain MaV203. Transformants were plated on SD medium lacking Trp and Leu and were replica-plated onto SD medium lacking Trp, Leu, and His and containing 10 mM 3-AT. In all cases, plates were photographed after 2 days in the dark at 30°C.

### Transcriptional and Transactivation Activity Assays in Rice Protoplasts

For transcriptional activity assays, the *OsKala3* and *OsKala4* ORFs were inserted in the place of *sGFP* into the p326-sGFP plasmid digested with *Xba*I and *Not*I to generate effector constructs. For the firefly luciferase (*fLUC*) reporter constructs, approximately 1- or 2-kb promoter regions of anthocyanin biosynthetic genes except for *OsUFGT* were PCR-amplified from genomic DNA isolated from rice cv. HN. PCR fragments for *OsCHS* (Os11g0530600), *OsCHI* (Os03g0819600), *OsF3H* (Os04g0662600), *OsF3’H* (Os10g0320100), *OsDFR* (Os01g0633500), and *OsANS* (Os01g0372500) were inserted into the p326-LUC vector. The reporter constructs also contained the renilla luciferase (*rLUC*) gene driven by the *UBIQUITIN10* (*UBQ10*) promoter as an internal control.

For transactivation assays, various *OsKala3* promoter fragments were PCR-amplified from rice cv. IM (one copy of the repeat unit [RU]) and HN (two RU copies) and cloned into the p326-LUC vector. Two additional reporter constructs (4 × and 8 ×) were generated by infusion with p326-2 × RU LUC vector digested with *Bam*HI and amplified PCR products by the primers listed in Supplementary Table 1. Isolation and transformation of rice protoplasts were performed as previously described (Kim et al., 2018). The firefly luciferase (LUC) and renilla luciferase (REN) was measured using a dual luciferase assay system (Promega, Madison, WI, United States) according to the manufacturer’s protocol. Normalized reporter activity was calculated from the LUC/REN ratio; this ratio was then set to 1 for transient transfection in the absence of effector.

### Identification of *OsKala3* Promoter Regions

Genomic DNA was extracted as previously described (Lim and Ha, 2013). The *OsKala3* promoter region was PCR-amplified from six rice cultivars (two white [IM and Dongjin (DJ)], two black [HN and HJJ], and two red [Hongjinju (HoJJ) and JJJ]) using the primers listed in Supplementary Table 1. The resulting PCR products were cloned into the pENTR-SD/D-TOPO vector (Invitrogen) for sequencing. Multiple sequence alignments were performed using ClustalW^1^. *Cis*-acting elements were predicted using the PlantCARE online tool^2^.

### Designing a Molecular Marker for Discriminating Rice Pericarp Color

Genomic DNA was extracted from the leaves of 40 rice varieties (13 white, 16 black, and 11 red). These varieties were categorized according to their pericarp color as follows: white: DJ, Hwayoung (HY), IM, Taichung 65 (T65), Hwasung (HwS), Saenuri (SNR), Nampyeong (NP), Shindongjin (SDJ), Kokushokuto-2 (A58), Milyang 252 (BR), Purple check (PC), Heugdaegu (HDG), and Jado (JD); black: Heughyang (HH), HJJ, Heugkwang (HK), HN, Heugseol (HS), Josengheugchal (JSHC), Pirurutong (PR), Suwon 493 (SW493), Suwon 505 (SW 505), Chunghyangheugkmi (CHHM), Joeunheugmi (JEHM), Sunhyangheugmi (SHHM), Nunkeunheugchal (NKHC), Nunkeunheugchal 1 (NKHC1), Boseokheugchal (BSHC) and Heugsujung (HSJ); and red: Aengmi (AM), Goryeong 8 (GR8), HoJJ, Hanyangjo (HYJ), JJJ, Jakwangdo (JKD), Siga-Chata Mochi (SCM), Susangjo (SSJ), Gunganghongmi (GGHM), Jeogjinju 2 (JJJ2), and Jeogjinjuchal (JJJC). PCRs were performed to amplify the cleaved amplified polymorphic sequence (CAPS) marker for *OsDFR* and the insertion/deletion (InDel) markers for *OsKala4* and *OsRc*, respectively, with previously reported primer pairs (Lim and Ha, 2013; Oikawa et al., 2015). An InDel marker was designed for *OsKala3* to score sequence variation in the *OsKala3* promoter. PCR conditions were as follows: initial denaturation at 98°C for 2 min followed by 30 cycles of 98°C for 10 s, 60°C for 15 s, and 68°C for 30 s. For CAPS analysis, PCR amplicons were digested with the *Taq*I restriction enzymes and electrophoresed on a 1.5% agarose gel. For InDel analysis, PCR products were resolved on a 3% agarose gel.

## Results

### Transcripts of *OsKala3* and *OsKala4* Genes Are Highly Expressed in Black Pericarp

**FIGURE 1:**
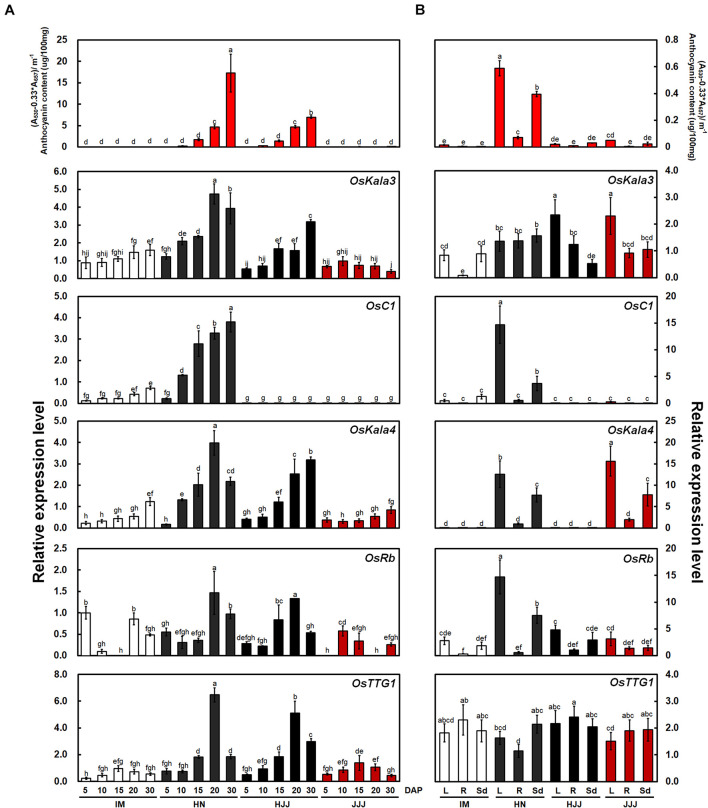
Anthocyanin contents and expression analysis of *OsKala3* and other putative anthocyanin regulatory genes from developing rice seeds and young seedling stage. **(A)** Mean anthocyanin accumulation in developing rice seeds from non-pigmented (IM), black (HN and HJJ), and red (JJJ) rice varieties during seed maturation (top). Relative transcript levels for regulators of anthocyanin biosynthesis (bottom). DAP, days after pollination. **(B)** Anthocyanin contents (top) and transcript level (bottom) of anthocyanin regulator genes in various tissues of the young seedlings stage (14 days after sowing). L, leaves; R, roots; Sd, seedlings. Results represent mean values ± SD from three independent biological replicates. Different letters above the bars indicate significantly different values (*p* < 0.05) calculated using two-way ANOVA followed by Duncan’s multiple range tests.

To investigate the relationship between MBW complexes and pericarp color, we analyzed the contents of anthocyanin and temporal expression of five genes (*OsKala3*, *OsC1*, *OsKala4*, *OsRb*, and *OsTTG1*) with rice seeds at the various developmental stages and vegetative tissues at the seedling stage (Figures 1A,B). Among R2R3 MYB TFs, *OsKala3* transcript levels were high in cultivars with black rice seeds such as HN and HJJ, while *OsC1* transcripts were detected only in the black-pigmented rice seeds of the HN cultivar. *OsKala4* was reported to be a positive regulator of anthocyanin accumulation in rice seed and showed an expression pattern similar to that of *OsKala3*, with high expression in cultivars HN and HJJ. The expression of *OsRb*, encoding another bHLH TF, was comparable across cultivars with all three colors of pericarp during seed development. *OsTTG1* transcript levels in the non-pigmented pericarp cultivar IM and the red pericarp cultivar JJJ were lower than those in the black pericarp cultivars HN and HJJ. Over the five stages of pericarp development sampled here, *OsKala3* expression was commonly expressed in cultivars with black pericarp and was closely linked to anthocyanin content and transcript level of anthocyanin biosynthetic genes (Supplementary Figure 1A). However, the expression level of *chalcone isomerase* (*OsCHI*) was similar in non-pigmented and black pericarp rice seeds. Notably, *OsC1* transcript levels were also correlated with the expression profile of most anthocyanin biosynthetic genes, but did not seem to be a common regulator on anthocyanin biosynthesis in rice pericarp, which was least expressed in the black pericarp rice cultivar HJJ. These results suggested that OsKala3, rather than OsC1, may play an important role in anthocyanin biosynthesis in rice pericarp.

In seedling plants, we confirmed the content of anthocyanin and expression levels with the same genes with above mentioned (Figure 1B). The transcript levels of *OsC1* was higher than those of *OsKala3*. Additionally, *OsC1* and *OsRb* transcript levels were higher than those of *OsKala3* and *OsKala4* in vegetative tissues. In addition, the expression profile of anthocyanin biosynthetic genes paralleled that of *OsC1* and *OsRb* in tissues accumulating anthocyanins, such as shoot and seedling from the HN cultivar (Supplementary Figure 1B). These results suggested that different TF combinations determine the precise spatio-temporal regulation of anthocyanin biosynthesis in rice.

### Phylogenetic Analysis of Anthocyanin Biosynthesis-Related MYB Domain Transcription Factors

**FIGURE 2:**
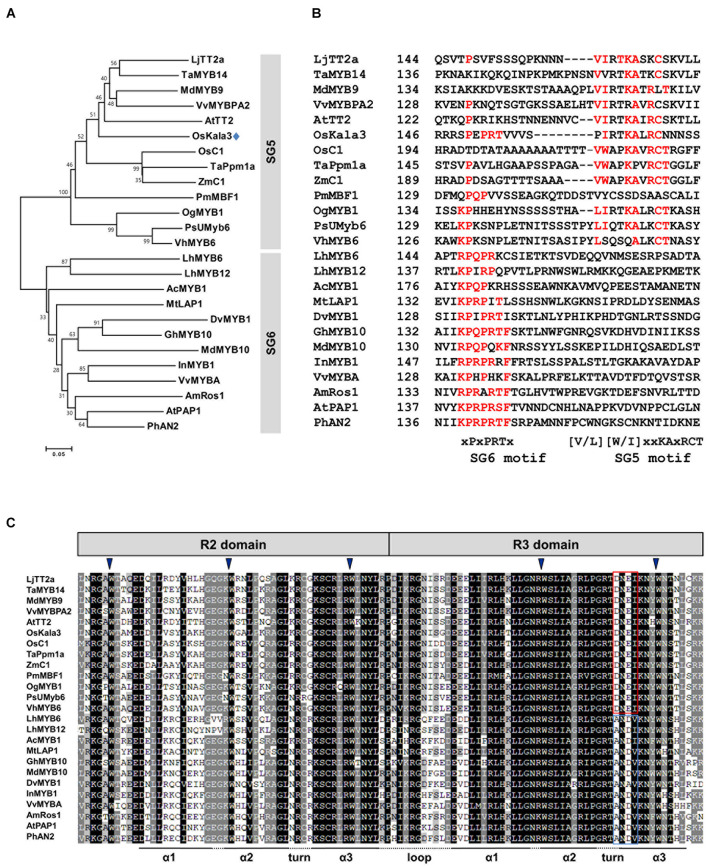
Phylogenetic relationships between anthocyanin biosynthetic regulators in rice and other species. **(A)** Phylogenetic tree of rice OsKala3 and R2R3 MYB proteins from other plants. The phylogenetic tree was constructed using the neighbor-joining method with MEGA6 software. GenBank database accession numbers are *Allium cepa* AcMYB1 (KX785130); *Antirrhinum majus* AmRos1 (ABB83826); *Arabidopsis thaliana* AtPAP1 (AAG42001), AtTT2 (CAC40021); *Dahlia pinnata* (syn. variabilis) DvMYB1 (AB601003); *Gerbera hybrida* GhMYB10 (CAD87010); *Ipomoea nil* InMYB1 (BAE94391); *Lilium hybrida* LhMYB6 (BAJ05399), LhMYB12 (BAJ05398); *Lotus japonicas* LjTT2a (AB300033); *Malus* × *domestica* MdMYB9 (ABB84757), MdMYB10 (ACQ45201); *Medicago truncatula* MtLAP1 (ACN79541); *Oncidium* Gower Ramsey OgMYB1 (ABS58501); *Oryza sativa* OsC1 (BAD04024), OsKala3 (BAA23339); *Petunia* × *hybrida* PhAN2 (AAF66727); *Phalaenopsis schilleriana* PsUMyb6 (FJ039860); *Picea mariana* PmMBF1 (U39448); *Trifolium arvense* TaMYB14 (AFJ53053); *Triticum aestivum* TaPpm1 (MG066451); *Vanda hybrida* VhMYB6 (ADQ57817); *Vitis vinifera* VvMYBA (BAD18977), VvMYBPA1 (AM259485); *Zea mays* ZmC1 (AAA33482). **(B)** Multiple sequence alignment of part of the C-terminal region of the R2R3-MYB sequences, showing the SG5 and SG6 motifs. Amino acids matching either the SG6 motif or the SG5 motif are indicated in red. The starting amino acid position of the sequences is given in the second column. **(C)** Multiple sequence alignment of the R2 and R3 domains across R2R3 MYB proteins shown in **(A)**. The conserved residues of DNEI and ANDV are represented using the red and blue boxes, respectively. Inverted blue triangles indicate the conserved residues forming the inner hydrophobic core of the R2 and R3 domains.

Previous studies suggested that flavonoid-related MYB-domain TFs belonging to the SG5 and SG6 subgroups of R2R3-MYB TFs regulate proanthocyanidin and anthocyanin biosynthesis in different tissues including leaves, fruits, and seeds (Stracke et al., 2001). In a phylogenetic tree of R2R3-MYB proteins from various plant species, we found that OsKala3 clustered into SG5 subgroup, together with OsC1, TaPpm1a, and ZmC1, which are monocotyledonous MYB proteins involved in anthocyanin biosynthesis as a positive regulator (Figure 2A; Jiang et al., 2018; Sun et al., 2018; Xia et al., 2021).

Several studies have reported that R2R3-MYB TFs from the SG5 and SG6 subgroup have the well conservative motifs of SG5 motif ([V/L][W/I]xxKAxRCT) and SG6 motif ([K/R]P[Q/R]P[Q/R]TF), respectively, in their C-terminal region (Stracke et al., 2001; Yamagishi et al., 2010). Notably, although OsKala3 belonged to the SG5 subgroup, it also contained a reasonably conserved SG6-like motif with the sequence xPxPRTx, which was clearly absent from other SG5-type R2R3 MYB TFs such as AtTT2, OsC1, and ZmC1 (Figure 2B). There is some precedent for this: in orchid species, the anthocyanin biosynthesis regulators OgMYB1, VhMYB6, and PsMYB6 share a weakly conserved SG6 motif in addition to a highly conserved SG5 motif, as in OsKala3. Similarly, onion (*Allium cepa*) AcMYB1 is a positive regulator of anthocyanin accumulation and possessed the SG6-like sequence xKPQPxxx. Sequence alignments showed that all R2R3-MYB TFs within the SG5 and SG6 subgroups share the conserved motif [D/E]Lx_2_[R/K]x_3_Lx_6_Lx_3_R in the R3 domain, which is functionally important for interaction between MYB and R/B-like bHLH proteins (Yamagishi et al., 2010). Additionally, the R2R3 domain of OsKala3 has the completely conserved five tryptophans that are important residue for forming the helix-loop-helix protein architecture. In the end of R3 domain, OsKala3 habored the absolutely conserved DNEI motif, characteristic SG5 subgroup (Figure 2C). Our results implied that OsKala3, like these MYB proteins from other species, belongs to the SG5 subgroup but also contains an SG6-like motif conferring a role in anthocyanin production.

### OsKala3, OsKala4, and OsTTG1 Are Nuclear Proteins

To explore the potential role of OsKala3, OsKala4, and OsTTG1 in transcriptional regulation, we fused each protein to green fluorescent protein (GFP). The resulting constructs were placed under the control of the cauliflower mosaic virus (CaMV) 35S promoter and transiently transfected in rice leaf protoplasts. The fluorescence pattern from control sGFP was distributed throughout the cell, including the nucleus and the cytoplasm (Supplementary Figure 2). We marked nuclei with red fluorescent protein (RFP) carrying a nuclear localization signal (NLS). When we transiently co-transfected the plants with this *35S:NLS-RFP* control, green fluorescence from *35S:OsKala3-GFP* and *35S:OsKala4-GFP* constructs clearly localized to the nucleus, while that from a *35S:OsTTG1-GFP* construct accumulated in both the nucleus and cytoplasm. These subcellular localization patterns were consistent with roles for these proteins in transcriptional activation.

### OsKala3, OsKala4, and OsTTG1 Form a Complex

**FIGURE 3:**
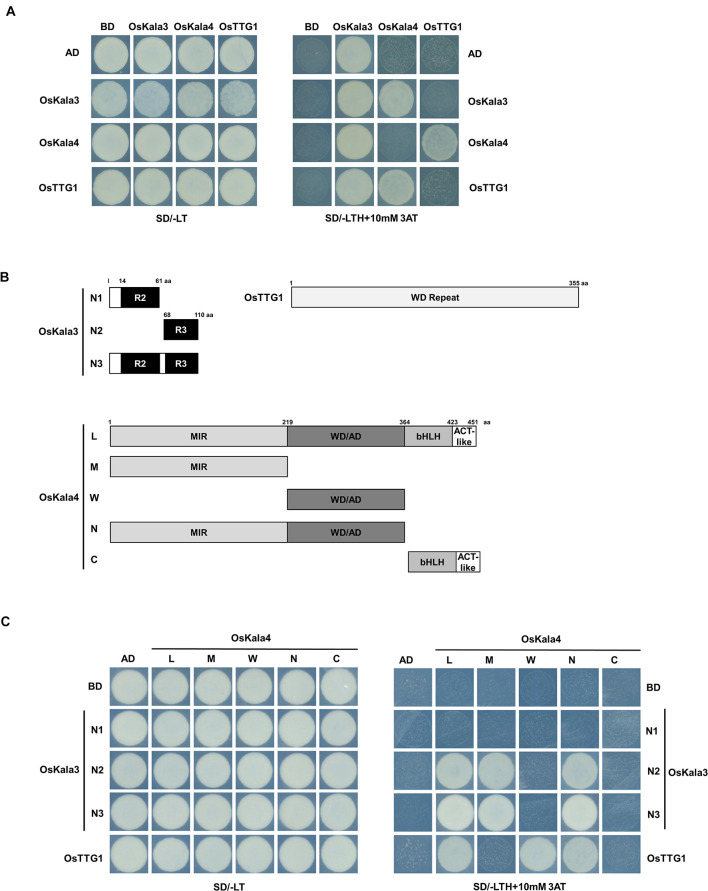
OsKala3, OsKala4, and OsTTG1 form a ternary complex. **(A)** Interactions between OsKala3, OsKala4, and OsTTG1 proteins, as revealed by yeast two-hybrid (Y2H) analysis. **(B)** Schematic representation of constructs used to delineate the interaction interface between OsKala3, OsKala4, and OsTTG1. Amino acid positions are indicated in the diagrams. **(C)** Interactions between truncated OsKala3, OsKala4, and full-length OsTTG1. AD, activation domain; BD, binding domain; SD, synthetic defined medium; 3AT, 3-amino-1,2,4-triazole; SD/–TL, minimal medium lacking Trp and Leu; SD/–TLH + 3AT, synthetic defined medium lacking Trp, Leu, His and containing 10 mM 3AT.

We next tested for interaction between OsKala3, OsKala4, and OsTTG1 in yeast two-hybrid (Y2H) assays. OsKala3 and OsKala4 appeared to physically interact, while OsTTG1 interacted only with OsKala4, but not OsKala3 (Figure 3A). Notably, full-length OsKala3 exhibited autoactivation when fused to the Gal4 DNA-binding domain. To define the domain within OsKala3 responsible for autoactivation, we generated five constructs corresponding to the full-length OsKala3 coding region and to truncated proteins containing the N-terminal (domains of R2, R3, and R2R3) or C-terminal regions of OsKala3 fused to GAL4-DB (Supplementary Figure 3). We detected strong autoactivation activity with full-length protein and the C-terminal region of OsKala3, but not with its N terminus alone, indicating that the C terminus of OsKala3 possesses transcriptional autoactivation activity.

To refine our knowledge of the interaction interface between OsKala3 and OsKala4, we tested the interaction potential between the N terminus of OsKala3 (R2, R3, and R2R3 fragments) fused to GAL4-DB and various OsKala4 domains fused to GAL4-AD, including full-length OsKala4, three N-terminal fragments (containing either the MYB interacting region (MIR), the WDR interacting region through the acidic domain (WD/AD), or both), and a C-terminal fragment corresponding to the bHLH and ACT domains (Figure 3B). Y2H demonstrated that the R3 domain of OsKala3 interacts with the MIR domain of OsKala4 (Figure 3C). Using the same OsKala4 constructs, we then explored the interaction potential between OsKala4 and OsTTG1. We found that the N-terminal WD/AD domain of OsKala4 was necessary and sufficient for interaction with OsTTG1. These results indicated that OsKala4 interacts with both OsKala3 and OsTTG1, although OsKala3 and OsTTG1 did not directly interact. We therefore hypothesized that OsKala4 serves as a bridge between OsKala3 and OsTTG1, underlining its importance in the formation of the MBW complex.

### The OsKala3-OsKala4-OsTTG1 Complex Regulates Transcription of Anthocyanin Biosynthetic Genes

Previous studies revealed a number of *cis-*elements, such as MYB-recognizing element (MRE) and bHLH-recognizing element (BRE), in the promoters of anthocyanin biosynthetic genes (Zhu et al., 2015) that determine promoter activity. Additionally, the distance between MRE and BRE was shown to contribute to the degree of promoter activation by the MBW complex. We therefore isolated the promoter regions from *chalcone synthase* (*CHS*), *CHI*, *flavanone 3-hydroxylase* (*F3H*), *flavonoid 3’-hydroxylase* (*F3’H*), *dihydroflavonol 4-reductase* (*DFR*), and *anthocyanidin synthase* (*ANS*) and analyzed them for the presence and number of *cis-*elements, as well as the distance between these elements (Supplementary Figure 4).

**FIGURE 4:**
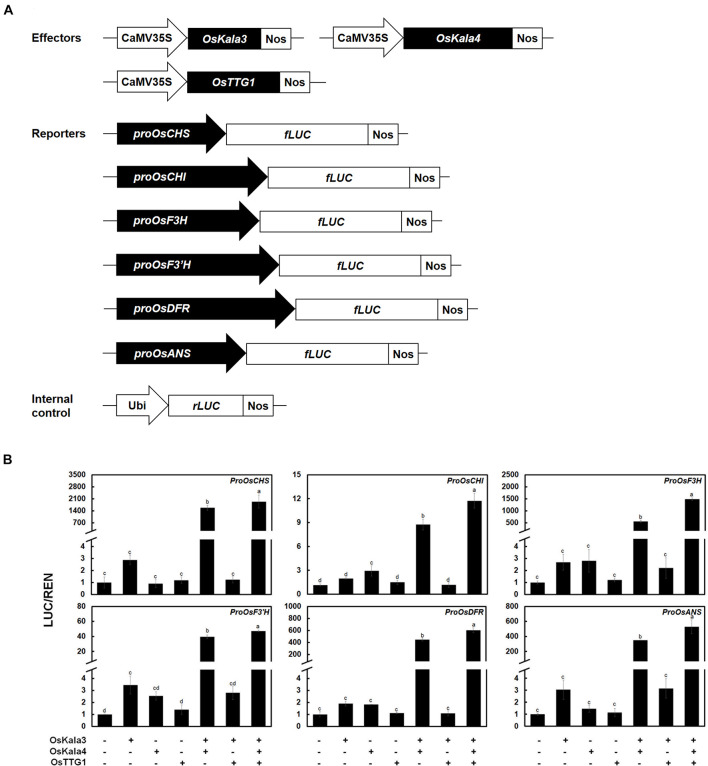
OsKala3, OsKala4, and OsTTG1 cooperate to induce the expression of anthocyanin biosynthetic genes. **(A)** Schematic representation of effector and reporter constructs used in the transcriptional activation assay. Effector constructs consist of the *OsKala3*, *OsKala4*, and *OsTTG1* open reading frames (ORFs) driven by the CaMV 35S promoter. In the reporter constructs, the firefly luciferase (*fLUC*) reporter gene is driven by the *OsCHS*, *OsCHI*, *OsF3H*, *OsF3’H*, *OsDFR*, or *OsANS* promoters. In the internal control constructs, the Renilla luciferase (*rLUC*) reporter gene is driven by the ubiquitin promoter. **(B)** Transactivation reporter assays showing the effects of OsKala3, OsKala4, and OsTTG1 on the transcription of anthocyanin biosynthetic genes. Results represent mean values ± SD from three independent biological replicates. Different letters above the bars indicate significantly different values (*p* < 0.0001) calculated using one-way ANOVA followed by a Duncan’s multiple-range test.

We then turned to the transcriptional activation potential on the promoters of anthocyanin biosynthetic genes by OsKala3, OsKala4, and OsTTG1 through a dual-luciferase assay in transiently transfected rice protoplasts (Figure 4A). We then normalized firefly luciferase (LUC) activity to renilla luciferase (REN) activity derived from a *UBQ10:rLUC* construct. The transient expression of *OsKala3* activated the transcription of anthocyanin biosynthetic genes to a higher level than that of *OsKala4* or *OsTTG1*, as determined by relative LUC activity (Figure 4B). Interestingly, transient co-transfection of *OsKala3* and *OsKala4* greatly increased the transcription of anthocyanin biosynthetic genes, by 11- to ∼548-fold relative to *OsKala3* alone, with the exception of the *proOsCHI:LUC* reporter, which produced a four-fold increase compared to *OsKala3* alone. By contrast, the transient transfection of *OsKala3* and *OsTTG1* resulted in transcriptional activation that was comparable to or slightly lower than that of *OsKala3* transfection alone. Co-transfection of all three components of the presumptive MBW complex also dramatically increased the promoter activity of anthocyanin biosynthetic genes, with luciferase activity often being even higher than that obtained when *OsKala3* and *OsKala4* were co-transfected without *OsTTG1* (Figure 4B). These results indicated that the expression of anthocyanin biosynthetic genes is induced by the OsKala3-OsKala4-OsTTG1 complex, implying that OsKala3 and OsKala4 work collaboratively to raise transcription of anthocyanin biosynthetic genes.

### Sequence Variation at *OsKala3* Is Associated With Anthocyanin Accumulation Resulting in Different Rice Pericarp Colors

**FIGURE 5:**
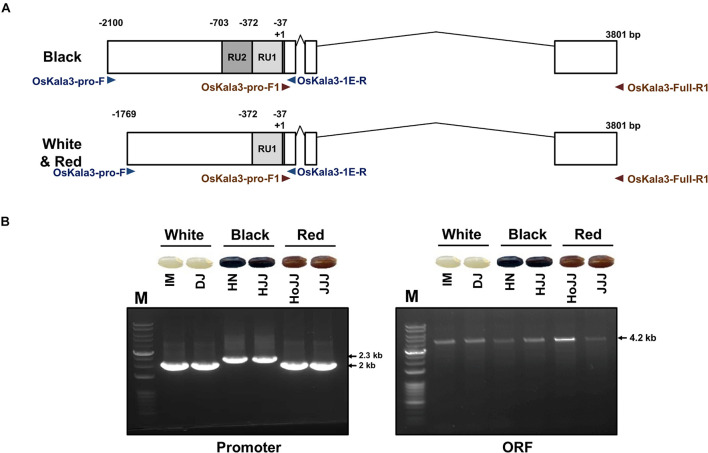
Validation of OsKala3 promoter variation. **(A)** Diagram of the genomic structure of OsKala3 from black, red, and white pericarp cultivars. The light gray box and dark gray box indicate repeat unit 1 (RU1) and RU2, respectively, in the *OsKala3* promoter region. **(B)** The amplicons’ representative gel image corresponds to promoter region (left) and ORF region (right). The primers used in this study are shown as arrows and listed in Supplementary Table 1.

We determined the sequence of the *OsKala3* coding regions and promoters in six rice cultivars with different pericarp colors (Figure 5A). PCR products amplified from the *OsKala3* promoter region of black pericarp cultivars were longer than those amplified from white and red pericarp cultivars. Sequencing revealed the nature of this insertion polymorphism: the *OsKala3* promoter contained two tandem repeat units (RUs) in all cultivars with black pericarp, each consisting of 335 and 331 bp, respectively. We designated this allele *OsKala3^2*xRU*^* (GenBank accession no. MW880722) (Figure 5B). Cultivars with white and red pericarp were characterized by only one RU (335 bp) in the *OsKala3* promoter region; their allele was designated *OsKala3^1*xRU*^* (GenBank accession no. MZ027587). The difference in RU number contributed to the size variation of the PCR products. This RU contained five seed-specific *cis*-elements and five MYB-binding *cis*-elements (Supplementary Figure 5), suggesting that *OsKala3* transcription levels may be higher in cultivars with black seeds relative to those with white or red seeds. Interestingly, the *OsKala3* coding region was identical among cultivars with white, red, and black pericarp, but the variation of *OsKala3* promoter region may contribute the expression level of *OsKala3* in seeds.

**FIGURE 6:**
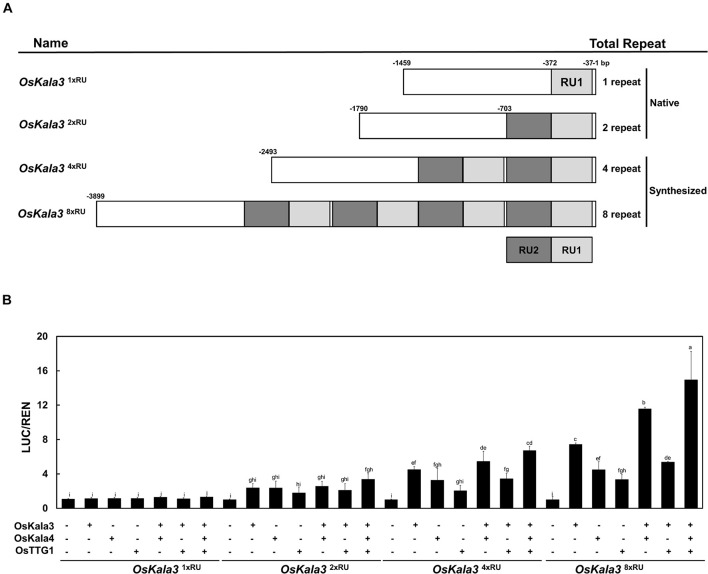
The number of repeat units in the *OsKala3* promoter region affects its transactivation rate. **(A)** Schematic representation of the promoter constructs used in the assay, bearing one, two, four, or eight repeat units (RU). The IM cultivar carries one RU copy in the *OsKala3* promoter, while the cultivar HJJ carries two copies of the element. RUs are indicated in light and dark gray. **(B)** Increasing the number of RU copies raises the activation of *OsKala3* transcription when co-transformed with *35S:OsKala3*, *35S:OsKala4*, and *35S:OsTTG1*. Results represent mean values ± SD from three independent biological replicates. The fLUC/rLUC ratio in the absence of effectors was set to 1.

Previous studies reported that insertion of RUs within the promoter region of transcriptional regulators can increase their promoter activity (Espley et al., 2009; Jiang et al., 2018). To quantify the effect of the RU detected within the *OsKla3* promoter on its expression levels, we transiently transfected reporter constructs driving the *LUC* reporter genes with different numbers of RUs (1, 2, 4, or 8) in rice protoplasts (Figure 6A). We observed a strong positive correlation between RU number and the activation of the reporter constructs when *OsKala3* was transiently transfected (Figure 6B). Indeed, although expression of *OsKala3* was not sufficient to activate the promoter harboring one RU, promoter activity gradually increased with rising RU numbers from RU2 to RU8, indicating that promoter transactivation is dependent on RU numbers. These results also confirmed that *OsKala3* can activate the native promoter from cultivars with black pericarp, as they harbor two RU copies, but not from cultivars with white or red pericarp, which have only one RU copy. These results indicated that RU number within the *OsKala3* promoter correlates strongly with the induction of *OsKala3* expression and forms a positive feedback loop with its own promoter.

We then tested the effect of transient transfection of *OsKala4* or *OsTTG1* on the transactivation potential of the same reporter constructs. Relative LUC activity increased over control levels when either *OsKala4* or *OsTTG1* was transfected into rice protoplasts, although activity levels were lower than those following transfection of *OsKala3* alone. Notably, co-transfection of *OsKala3* and *OsKala4* resulted in stronger activation of the chimeric promoters. Indeed, all the promoters from RU2 to RU8, except for RU1, were activated by *OsKala3*, and they were somewhat enhanced with the addition of *OsKala4*, although the pattern of increased activation depending on the RU number remained the same. In this assay, even RU8 activity was significantly enhanced with the combination of OsKala3 and OsKala4 proteins. It indicated that the level of promoter activity was dramatically increasing due to the presence of the number of RU sequences.

### Discrimination of Black Rice Pericarp by the Sequence Variation at the *OsKala3* and *OsKala4*

**FIGURE 7:**
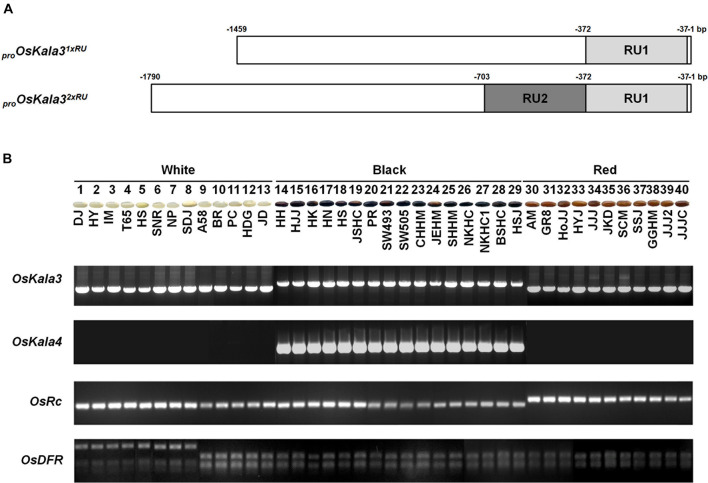
Variation at *OsKala3*, *OsKala4*, *OsRc*, and *OsDFR* predicts pericarp color across 40 rice cultivars. **(A)** Schematic representation of *OsKala3* promoter region. The light gray box and dark gray box indicate RU1and RU2, respectively, in the *OsKala3* promoter region. The position of the RU is indicated relative to the ATG translation start site. **(B)** Representative photographs of rice seeds showing pericarp colors for the indicated cultivars (top). Genotyping results of the 40 rice cultivars with the and InDel markers designed against *OsKala3*, *OsKala4*, and *OsRc* and CAPS marker designed against *OsDFR* (bottom).

To explore the relationship between the number of RUs of OsKala3 promoter region and pericarp color phenotype, we genotyped 40 rice cultivars with different pericarp colors using specific markers developed in this study for *OsKala3* and previously reported for *OsKala4 OsRc*, and *OsDFR* (Lim and Ha, 2013; Oikawa et al., 2015). We found that all cultivars with black pericarp shared the *OsKala3^2*xRU*^* genotype, while the *OsKala3^1*xRU*^* allele was common to cultivars with white and red pericarp (Figure 7). In addition, all cultivars with black pericarp shared the same *OsKala4* alleles that harbored two RU copies in the promoter region, while all cultivars with white and red pericarp have only one RU copy, as expected. As in the previous studies, we verified that the red seed cultivars had the functional allele of *Rc*, but all cultivars with black and white cultivars had the shorter fragment of *Rc*. Also, all cultivars of black and red pericarp had the functional allele of DFR, but some of white cultivars had the mutation led to premature stop codon. Taken together, these results revealed the transcript level of *OsKala3* and *OsKala4* due to their promoter variation are closely associated with pericarp color in rice and functional DFR is a cornerstone for pericarp pigmentation.

## Discussion

We discovered here that an insertion in the promoter region of the gene encoding the R2R3 MYB TF OsKala3 contributes to higher *OsKala3* expression in seeds, resulting in black pericarp as a consequence of anthocyanin accumulation. Deciphering the regulatory mechanism behind anthocyanin biosynthesis and its spatio-temporal control has significant implications for the development of novel plant and fruit varieties with higher anthocyanin contents through either conventional or advanced breeding methods.

### OsKala3 and OsKala4 Collaboratively Activate Anthocyanin Biosynthesis in Pericarp

Among ternary MBW complex, the expression of genes encoding MYB or bHLH TFs has been shown to control the site and timing of anthocyanin biosynthesis (Yamagishi et al., 2010; Yuan et al., 2014). The activity of MYB determines the amount of anthocyanin, contributing for different color intensities with co-factor bHLH TFs. The bHLH TFs in MBW complex activate the anthocyanin biosynthesis dependent on available partners present in the specific cell types at particular stage (Hichri et al., 2011).

Previous studies revealed that *OsKala4* (encoding a bHLH TF) was a tissue-specific regulator for anthocyanin biosynthesis in rice pericarp, as exhibiting the higher expression level due to the rearrangement of its promoter region (Oikawa et al., 2015). However, seed-specific anthocyanin activating R2R3 MYB TF was largely unknown until now. We found that during seed development, *OsKala3* (encoding an MYB TF) was highly expressed in the rice cultivars HN and HJJ, which are characterized by black pericarp. However, previously reported another R2R3 MYB TF, *OsC1*, was highly expressed in HN seeds, but extremely low expression levels in HJJ seeds. Several studies reported that OsC1 was a color-producing gene via interaction with counterpart bHLH TFs and accumulated anthocyanin production in leaf sheath, apiculus, and hull, but not in seeds (Fan et al., 2008; Chin et al., 2016; Sun et al., 2018). Meng et al. (2021) verified that OsC1 itself was not able to activate the transcription of anthocyanin biosynthetic genes but can produce anthocyanin when co-expression of OsPa, an apiculus-specific bHLH TF. These results speculate that anthocyanin biosynthesis positively regulated by the transcript of MYB and bHLH TFs present in the cells at given developmental stage.

**FIGURE 8:**
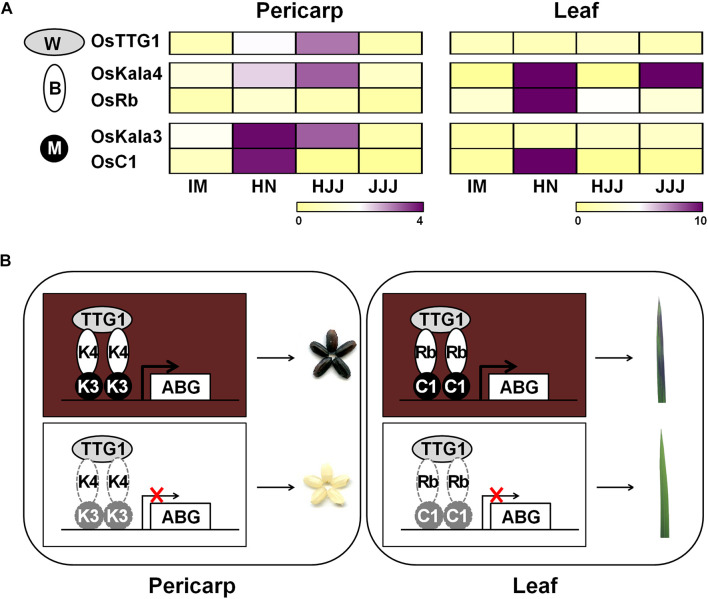
Proposed model of anthocyanin biosynthesis of seed and vegetative tissue in rice. **(A)** Heat map diagram of expression level for anthocyanin regulators consisting of MYB (M), bHLH (B), and WD40 (W) from rice pericarp and leaf, respectively. Color scale indicates fold changes in gene expression. **(B)** Working models of anthocyanin coloration in different tissues of rice. Both *OsKala3* and *OsKala4* had extremely high expression levels in rice pericarp, while *OsC1* and *OsRb* were more strongly expressed in respective rice leaf. When all of OsDFR was functional, it forms the functional MBW complex consisting of OsKala3-OsKala4-OsTTG1 and OsC1-OsRb-OsTTG1 activating anthocyanin biosynthesis in rice.

During seed developmental stages, the transcript level of *OsKala3* and *OsKala4* were highly expressed in the black pericarp cultivars HN and HJJ. In addition, these expression patterns persisted across developmental stages (Figure 1). In the HN cultivar, *OsKala3* and *OsKala4* transcript levels gradually increased, reaching their peak at 20 days after pollination (DAP), followed by a slight drop at 30 DAP. In the case of the HJJ cultivar, *OsKala3* and *OsKala4* transcripts gradually increased until 30 DAP. Another MYB TF-encoding gene, *OsC1*, was expressed in HN seeds, but not in HJJ seeds. Analysis of expression profiles in different tissues showed that *OsKala3* transcripts were abundant in seeds with black pericarp (Figure 1A), while *OsC1* was predominantly expressed in vegetative tissues, including shoots and young seedlings. Taken together, it suggests that *OsKala3*, but not *OsC1*, exhibit the appropriate expression pattern to regulate the expression of anthocyanin biosynthetic genes and anthocyanin contents in seeds. Also, previous studies reported that tissue-specific expression of bHLH TFs encoding *OsKala4* and *OsRb* exhibits the different pigmentation pattern (Zheng et al., 2019). Our results showed that expression pattern of *OsKala3* and *OsKala4* was well consistence of anthocyanin contents in pericarps. Among the component of MBW complex, transcript of OsTTG1 was constitutively detected in all tissues tested but its role was still unknown. Y2H assays demonstrated that OsKala3 interacts only with OsKala4, while OsKala4 interacted with OsTTG1, possibly acting as a bridge between the partners of the MBW complex (Figure 3). We refined the interaction domains to the R3 domain of OsKala3 and the MIR domain at the N terminus of OsKala4, while OsTTG1 interacted with the WD/AD region at the N terminus of OsKala4. These results highlight the importance of the OsKala4 N-terminal region for interaction with both OsKala3 and OsTTG1. In addition, we determined that the transcription of anthocyanin biosynthetic genes was induced by transfection with *OsKala3* alone in rice protoplasts, but co-transfection of *OsKala4* and *OsTTG1* with *OsKala3* dramatically increased transcriptional activity (Figure 6). It concluded that OsTTG1 was a significant factor for full activation of anthocyanin biosynthetic genes by interacting with OsKala3 and OsKala4. Based on these results, we proposed a model of anthocyanin biosynthesis in seed and vegetative tissue of rice (Figure 8). The OsKala3-OsKala4-OsTTG1 and the OsC1-OsRb-OsTTG1 gene systems regulates the anthocyanin biosynthesis in a tissue-specific manner. Thus, the spatial and temporal interaction between R2R3-MYB TFs (OsKala3 or OsC1) and bHLH TFs (OsKala4 or OsRb) activate and elevate the expression levels of anthocyanin biosynthetic genes, resulting in color development in various tissues including pericarp or leaf, respectively.

### The Number of Repeat Units in the *OsKala3* Promoter Is Important for Transcriptional Activation

Interestingly, the OsKala3 coding region was identical among cultivars with white, red, and black pericarp, but the *OsKala3* promoter region of cultivars with black pericarp carried two RUs instead of the typical single RU found in cultivars with white or red pericarp (Figure 7). This RU contained *cis*-elements for seed-specific and MYB binding (Supplementary Figure 5), suggesting that the higher transcript level of *OsKala3* was responsible for black pericarp.

Transcription factors bind to *cis*-elements within promoter regions to modulate gene expression, making the identification of *cis*-elements in promoters critical to deciphering the network of TFs and their target genes. Sometimes, the target site of a TF may also be present in the promoter region of its encoding gene, resulting in an auto-regulated loop. For instance, comparison of the proximal promoter region of apple (*Malus domestica*) *MdMYB10* showed that apple trees with white-fleshed fruits and green foliage have one copy of a 23-bp sequence (the R1 motif) (Espley et al., 2009). In contrast, red fleshed and red foliage apple trees contain six copies of the sequence (referred to as the R6 motif), five of which form a minisatellite-like polymorphism crucial for modulating transactivation levels of the gene. Mutating or deleting this repeat sequence led to a significant reduction in MdMYB10 transactivation by hindering autoregulation. Similarly, the expression of pear (*Pyrus communis* L.) *PcMYB10* and Arabidopsis *PRODUCTION OF ANTHOCYANIN PIGMENT1* (*PAP1*) may be increased when the R6 motif is inserted within their promoter regions, resulting in strong autoactivation and elevated anthocyanin accumulation (Brendolise et al., 2017). In monocots, wheat (*Triticum aestivum*) cultivars with white pericarp are characterized by a single copy of a 261-bp sequence in the proximal promoter region of purple pericarp-bHLH 1 (*TaPpb1*), but the purple-pericarp wheat cultivar H76 contains six copies organized as tandem repeats (Jiang et al., 2018). As shown in other plant species, variation in the promoter region of TF-encoding genes contributes to differential anthocyanin accumulation; furthermore, additional copies of a regulatory element can elevate transcription rates. In this study, we discovered that OsKala3 differed between cultivars with white, red, and black pericarp (Figure 4): cultivars with black pericarp carried two tandem RUs in the *OsKala3* promoter, while those with white or red pericarp had only one copy. Moreover, the presence of more copies of the RU in chimeric *OsKala3* promoters significantly increased transcription output (Figure 6). In addition, OsKala3 formed a positive feedback loop for its expression, but only in cultivars with black pericarp and two copies of the RU. Thus, the variation in the number of RUs among cultivars may affect the activation of *OsKala3* expression.

## Conclusion

In this study, we characterized OsKala3 as a common key regulator of pericarp anthocyanin pigmentation acting through the formation of a complex with OsKala4. The variation in RU number at the *OsKala3* promoter may affect anthocyanin accumulation by modulating *OsKala3* expression levels. OsKala3 and OsKala4 have similar spatial and temporal expression profiles, as they are highly expressed in pericarp and collaboratively regulate the expression of anthocyanin biosynthetic genes. These findings reveal the molecular and genetic basis of anthocyanin production in rice pericarp and should help facilitate sophisticated anthocyanin enhancement in pericarp.

## Data Availability Statement

The datasets presented in this study can be found in online repositories. The names of the repository/repositories and accession number(s) can be found in the article/Supplementary Material.

## Author Contributions

S-HL and J-YL devised the study and wrote the manuscript. D-HK and JY generated the constructs and performed the molecular experiments. JKK and S-HH contributed to the conceptual framework. All authors contributed to the article and approved the submitted version.

## Conflict of Interest

The authors declare that the research was conducted in the absence of any commercial or financial relationships that could be construed as a potential conflict of interest.

## Publisher’s Note

All claims expressed in this article are solely those of the authors and do not necessarily represent those of their affiliated organizations, or those of the publisher, the editors and the reviewers. Any product that may be evaluated in this article, or claim that may be made by its manufacturer, is not guaranteed or endorsed by the publisher.

## Footnotes

1
http://www.ebi.ac.uk

2
http://bioinformatics.psb.ugent.be/webtools/plantcare/html/
