# Supplementary material for: An OsKala3, R2R3 MYB TF, Is a Common Key Player for Black Rice Pericarp as Main Partner of an OsKala4, bHLH TF
Source: Front Plant Sci. 2021 Oct 29;12:765049. doi: 10.3389/fpls.2021.765049 (PMC8585765; doi:10.3389/fpls.2021.765049)
Supplement: Supplementary file 1 [file Data_Sheet_1.docx]

**Supporting information for Journal of Experimental Botany**

**An OsKala3, R2R3 MYB TF, is a common key player for black rice pericarp as main partner of an OsKala4, bHLH TF**

Da-Hye Kim^1,2^, JuHee Yang^2^, Sun-Hwa Ha^3^, Jae Kwang Kim^4^, Jong-Yeol Lee^2^* and Sun-Hyung Lim^1*^

**MATERIALS AND METHODS**

**RNA extraction and RT-qPCR**

Total RNA was extracted from developing rice seeds at various developmental stages and vegetative tissues at the seedling stages using the Fruit-mate for RNA Purification solution (Takara, Otsu, Japan) and Plant RNA Purification Reagent (Invitrogen, Carlsbad, CA, USA) as described previously (Kim *et al*., 2018) and then purified using the FavorPrep™ Plant Total RNA Mini Kit (Favorgen, Changzhi, Taiwan). First-strand cDNAs were synthesized from 2 μg of total RNA using amfiRivert cDNA Synthesis Platinum Master Mix (GenDEPOT, Barker, TX, USA). qPCRs were performed using AccuPower 2x Greenstar qPCR Master Mix (Bioneer, Daejun, Korea) and a Bio-Rad CFX96 Detection System (Bio-Rad Laboratories, Hercules, CA, USA), according to the manufacturer’s instructions. The expression levels of all target genes were normalized to that of *Ubiquitin* (*OsUBI*) as an internal reference. Gene-specific primers used for qPCR analysis are listed in Supplementary Table S1. Three independent biological replicates and three technical replicates were performed for each sample.

**Subcellular localization analysis**

For subcellular localization analysis, the open reading frames (ORFs) of *OsKala3* (BAA23339), *OsKala4* (AB021080), and *OsTTG1* (KAB8088430) from rice cv. HN were PCR-amplified with PrimeSTAR® HS DNA Polymerase (Takara) using gene-specific primer sets (p326-OsKala3-F/R, p326-OsKala4-F/R, and p326-OsTTG1-F/R) and cloned into the p326-sGFP plasmid linearized by *Xba*I digestion using the In-Fusion HD Cloning Kit (Takara). The resulting plasmids encoding C-terminal GFP fusion constructs were sequenced for confirmation of error-free PCR amplification. The plasmids were then introduced into rice protoplasts prepared from rice leaves using a polyethylene glycol (PEG)-mediated transformation procedure, as described by Kim *et al* (2018). The accumulation of the fusion proteins (OsKala3-GFP, OsKala4-GFP, and OsTTG1-GFP) was determined 16–20 h after transfection, and images were captured by confocal laser scanning microscopy (Leica TCS SP8; Leica Microsystems, Wetzlar, Germany).

**Transactivation Assays**

To generate the OsKala3 BD constructs, complete and partial regions of the OsKala3 coding sequence were individually amplified using specific primer sets (Supplementary Table S1). The amplified fragments were cloned into pGBKT7 vectors harboring the GAL4 DNA-binding domain (Takara) using an In-Fusion HD Cloning Kit (Takara). The individual BD constructs were transformed into the yeast strain AH109, following the manufacturer’s instructions (Takara). The transformed yeast cells were grown on SD media lacking Trp and were replicated on SD media lacking Trp, His, and Ade containing X-α-gal for color development. After two days in darkness at 30 °C, the plates were photographed.

**Sequence analysis**

To isolate the promoter region of anthocyanin biosynthetic genes, we amplified the approximately 1- or 2-kb promoter regions for *OsCHS* (Os11g0530600), *OsCHI* (Os03g0819600), *OsF3H* (Os04g0662600), *OsF3'H* (Os10g0320100), *OsDFR* (Os01g0633500), and *OsANS* (Os01g0372500) by PCR with genomic DNA isolated from rice cv. HN. The resulting PCR products were cloned into the pENTR-SD/D-TOPO vector (Invitrogen) and validate their sequencing. Cis-acting elements of each promoter were predicted using the PlantCARE online tool (http://bioinformatics.psb.ugent.be/webtools/plantcare/html/).

**Analysis of OsKala3 promoter**

To compare the OsKala3 promoter region among six cultivars (2 white, 2 black and 2 red), we performed the PCR with genomic DNAs and analyzed the amplified fragments. To analyzed the sequence of OsKala3 promoter region, we cloned the PCR resultant into the pENTR-SD/D-TOPO vector (Invitrogen) for validating their sequencing. Multiple sequence alignments were performed by ClustalW (<http://www.ebi.ac.uk>)

**
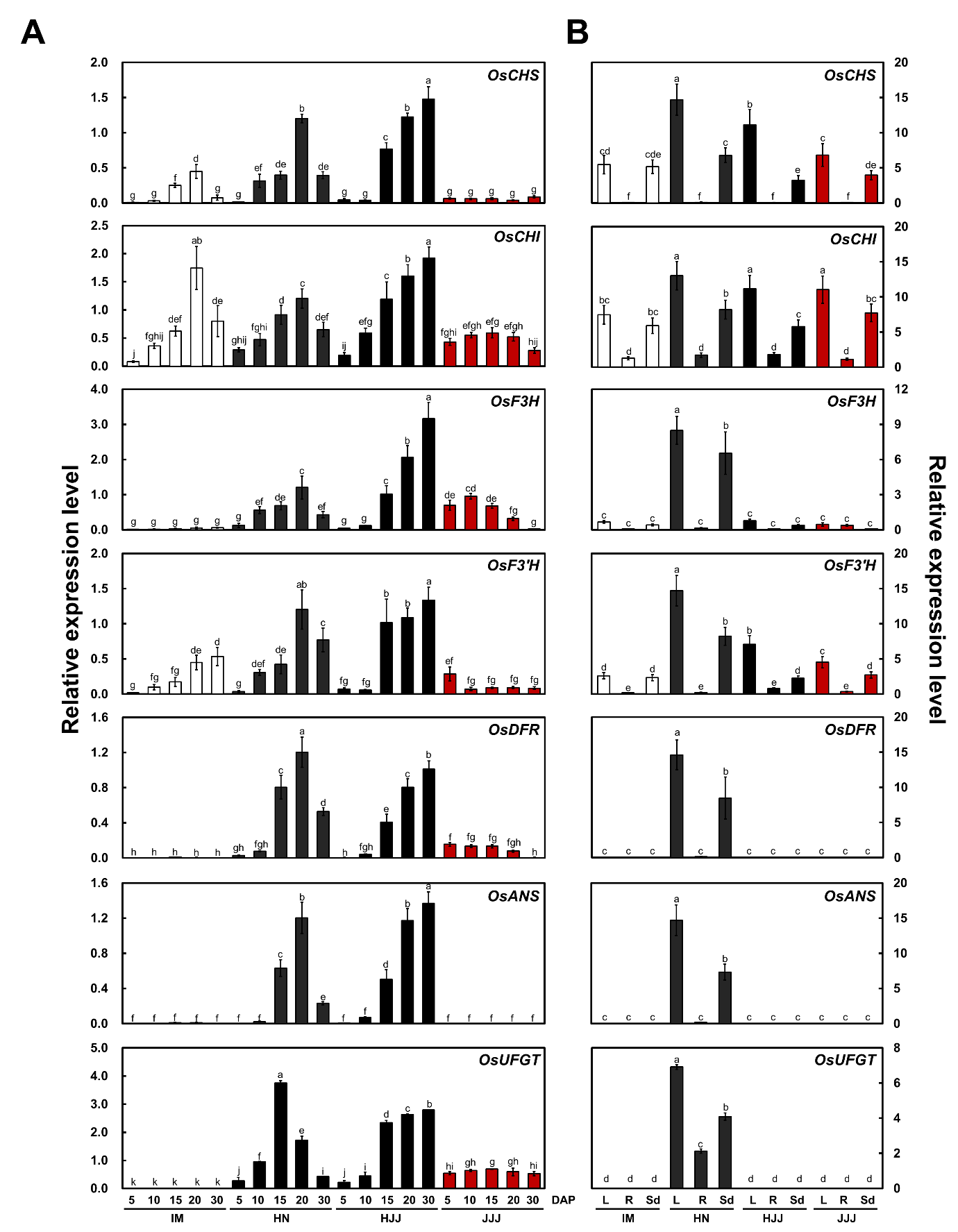
Supplementary Fig. S1.** Expression of anthocyanin biosynthetic genes in developing rice seeds from nonpigmented (IM), black (HN and HJJ), and red (JJJ) rice varieties **(A)** and in the young seedlings (14 days after sowing) including leaves (L), roots (R), and seedlings (Sd) (**B**). DAP, days after pollination. All transcript levels were measured in three independent biological replicates. *OsUBI* was used as reference. Data are shown as mean ± standard error (SE) from three independent biological replicates. Different letters above the bars indicate significantly different values (*P* < 0.05, two-way ANOVA followed by Duncan’s multiple range test).

**
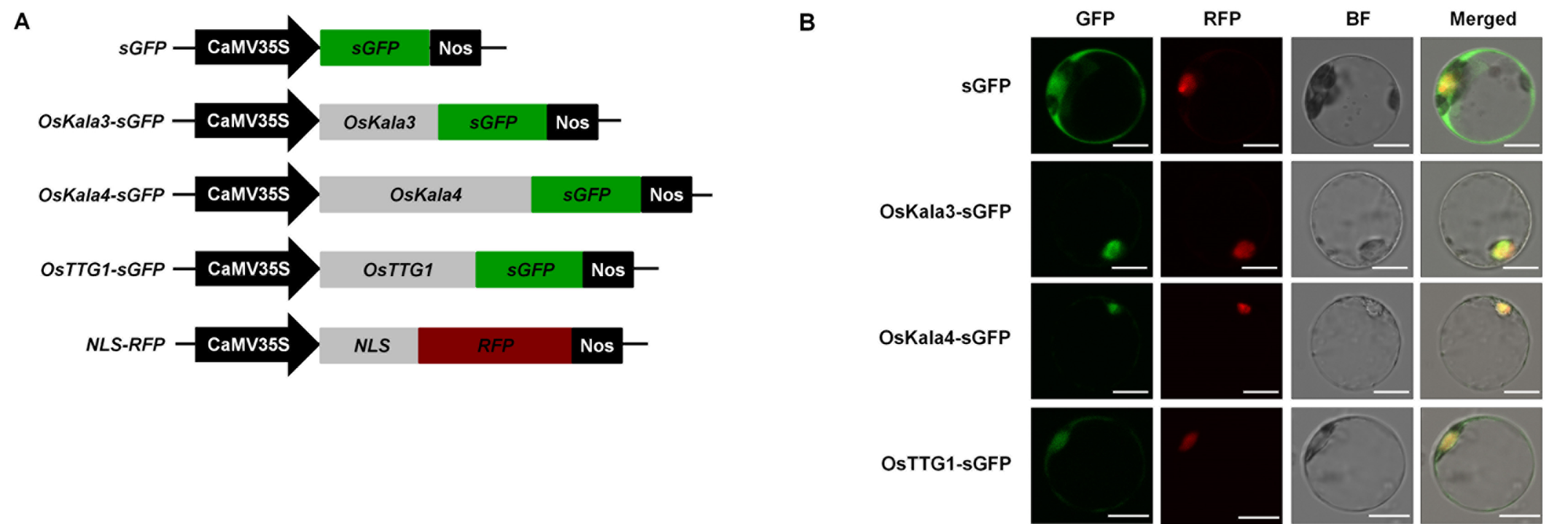
**

**Supplementary Fig. S2**. Subcellular localization of OsKala3, OsKala4, and OsTTG1 in rice leaf protoplasts.

(**A**) Schematic representation of the constructs used in this experiment: *sGFP*, soluble GFP; *OsKala3:GFP*, OsKala3 fused to GFP; *OsKala4:GFP*, OsKala4 fused to GFP; *OsTTG1:GFP*, OsTTG1 fused to GFP; and *NLS:RFP*, nuclear localization signal fused with RFP. (**B**) OsKala3, OsKala4, and OsTTG1 localize to the nucleus in rice leaf protoplasts. Microscopy visualization was performed 16 h after transformation. Bar = 10 μm.

**
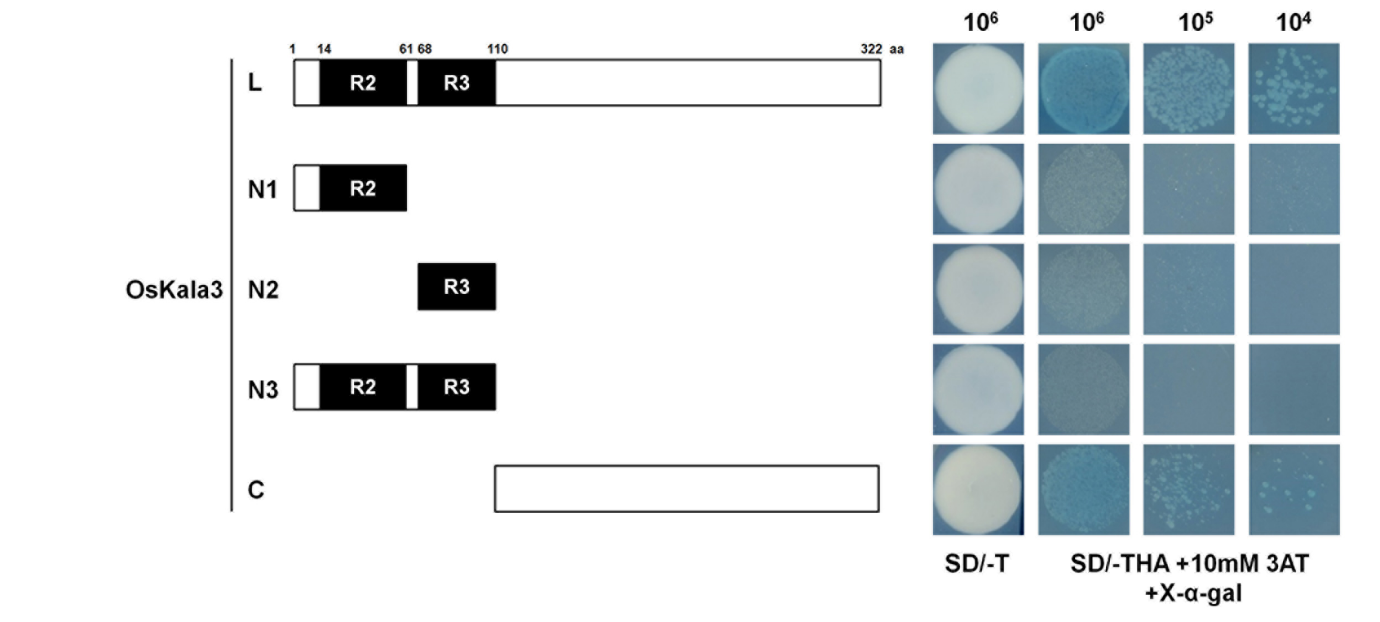
**

**Supplementary Fig. S3.** Transcription autoactivation assay with various OsKala3 domains. The full-length *OsKala3* ORF, the R2 domain region, the R3 domain region, the R2R3 MYB domain-containing N-terminal region, and the C-terminal region were fused to the GAL4 DNA-binding domain from pGADT7. Transformants were selected on synthetic defined medium lacking histidine (His) and including 3-AT at the indicated concentration. Amino acid positions are indicated in the diagrams. SD/−T, synthetic defined medium lacking Trp; SD/-THA, synthetic defined medium lacking Trp, His and Ade, 3AT, 3-amino-1,2,4-triazole.

**
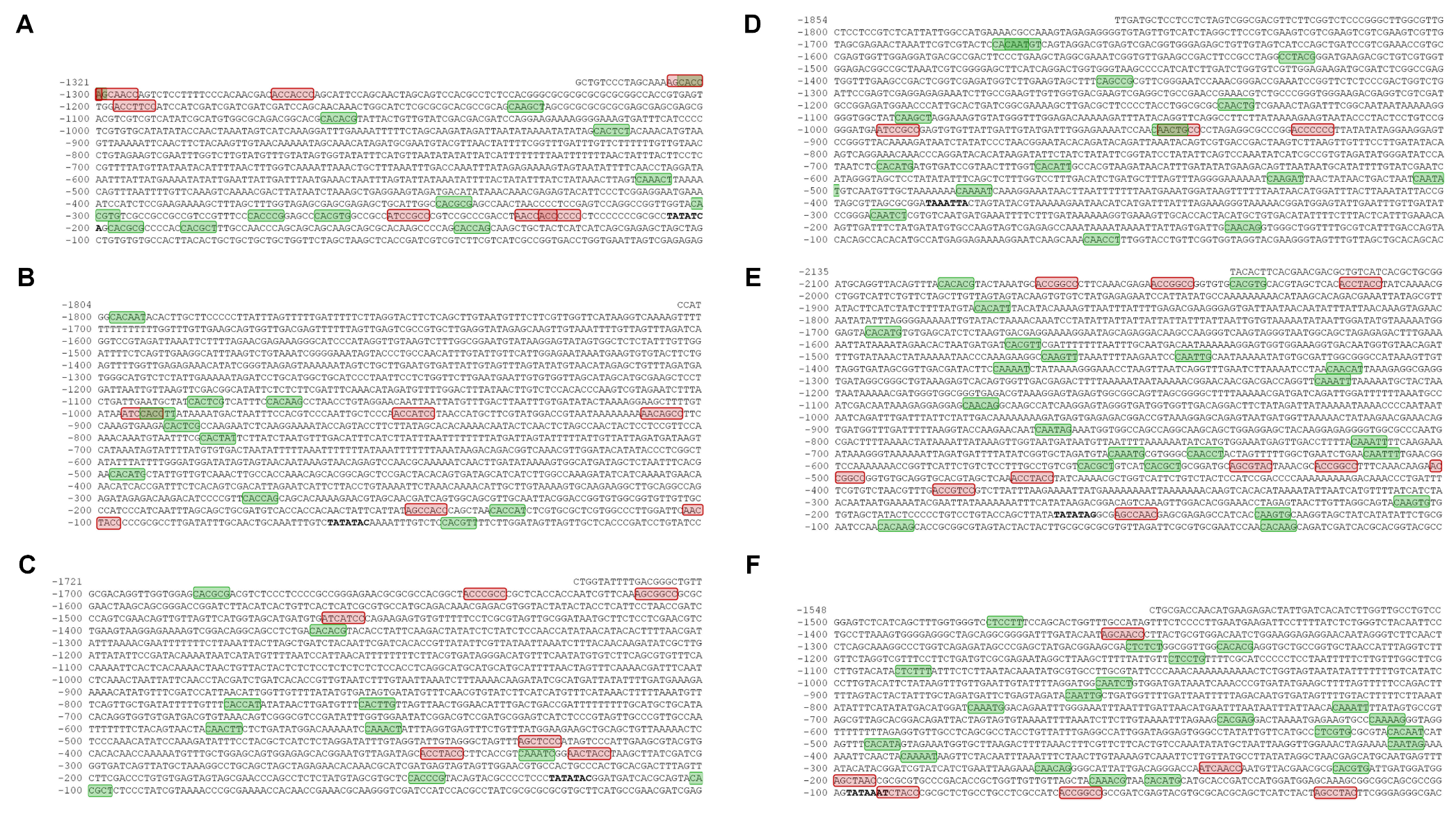
 Supplementary Fig. S4.** Nucleotide sequences of *OsCHS*, *OsCHI*, *OsF3H*, *OsF3'H*, *OsDFR*, and *OsANS* promoter.

Putative *cis*-elements bound by bHLH and MYB-domain transcription factors involved in anthocyanin biosynthetic genes. Partial promoters of *OsCHS* **(A)**, *OsCHI* **(B)**, *OsF3H* **(C)**, *OsF3'H* **(D)**, *OsDFR* **(E)**, and *OsANS* **(F)** genes. MYB-recognizing elements (MREs) and bHLH-recognizing elements (BREs) are indicated by red and green boxes, respectively, and the predicted TATA box is shown in bold.

**
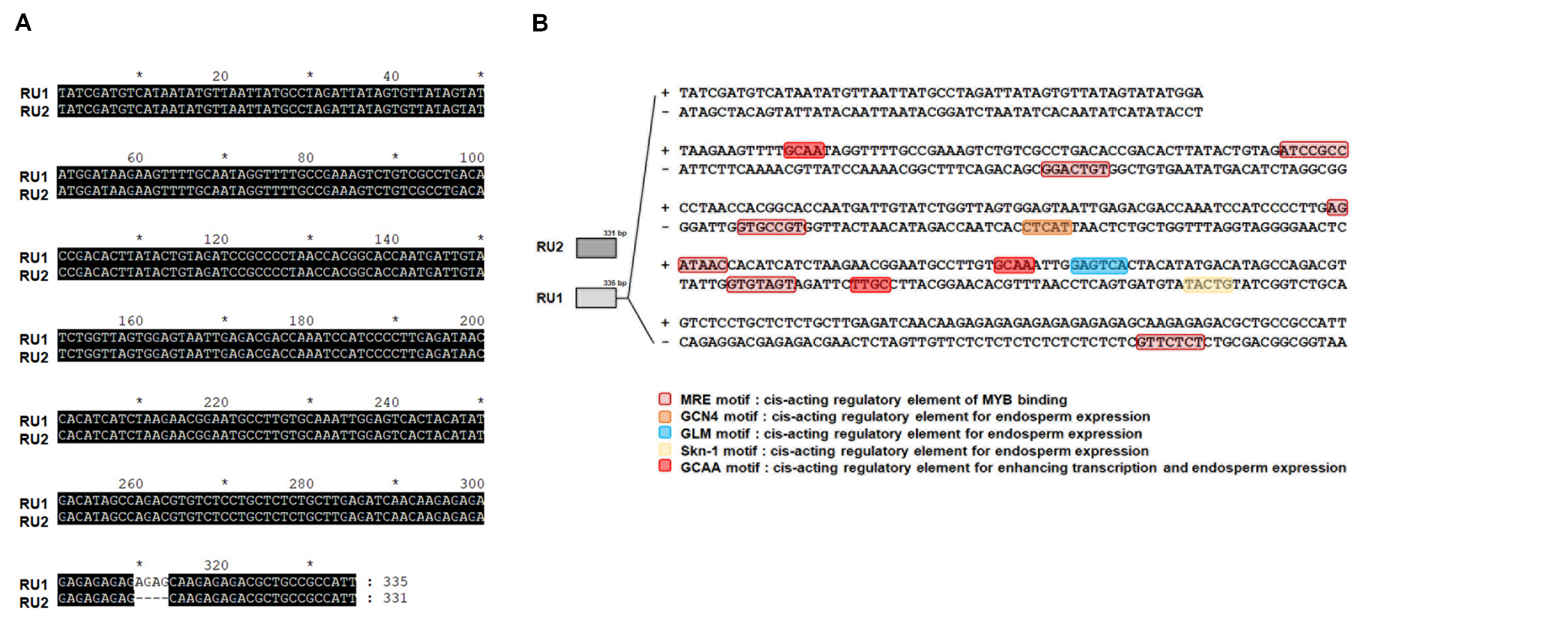
**

**Supplementary Fig. S5.** Characterization of RU in *OsKala3* promoter.

(**A**) Multiple sequence alignment of the RU1 and RU2 regions. RU1 is 4 bp longer than RU2. (**B**) *cis*-element analysis of the RU1 region. Seed-specific elements and MYB-recognizing elements (MREs) are indicated by various colored boxes, respectively.

**Supplementary Table S1*.*** List of primers used in this study.

| Usage | Primer name | Primer sequence |
| --- | --- | --- |
| Gene expression | qRT-OsKala3-F | CACCACCAGTAGGAGGAGGA |
|  | qRT-OsKala3-R | TTCGATTCCACAATGATCCA |
|  | qRT-OsC1-F | CTGGAACTTGTGTGCGTGAC |
|  | qRT-OsC1-R | CCCGCAACTGCACTTAAAAT |
|  | qRT-OsKala4-F | CCGAGAGAAGCTCAACGAGA |
|  | qRT-OsKala4-R | TGCAAGTATGGATGCCTTGT |
|  | qRT-OsRb-F | TCAAGAGCCTCCGTCTTGAT |
|  | qRT-OsRb-R | AATTAACGCCACAACGCTCT |
|  | qRT-OsTTG1-F | CAGCTTACCCGGAGTGGATA |
|  | qRT-OsTTG1-R | CCAAGGCTTCAGAGTCGAAC |
|  | qRT-OsCHS-F | GGGCTCATCTCGAAGAACAT |
|  | qRT-OsCHS-R | CCTCATCCTCTCCTTGTCCA |
|  | qRT-OsCHI-F | AATCGAGCTGCGAATTAACC |
|  | qRT-OsCHI-R | CGCGATTTCTCCTTTCCTTT |
|  | qRT-OsF3H-F | AGCACAGAAGCCCAAGTCTC |
|  | qRT-OsF3H-R | CTTCGATTTTCGACGGAAGA |
|  | qRT-OsF3'H-F | ACGGATTCATCAACGAAAGG |
|  | qRT-OsF3'H-R | AGCAGCACGCTTAGAAGGTC |
|  | qRT-OsDFR-F | GCGAGAAGGAACCGATACTG |
|  | qRT-OsDFR-R | TCCAAATCTCGCATTGTGAA |
|  | qRT-OsANS-F | GCATCGAACGGAATGAGAAC |
|  | qRT-OsANS-R | TTCGCTTCCGTTGAACATTA |
|  | qRT-OsUFGT-F | GGGCGTCGGTGATGGAGG |
|  | qRT-OsUFGT-R | ATGCACAGATTATCTCGACGAACT |
|  | qRT-OsUBI-F | GAAGTAAGGAAGGAGGAGGA |
|  | qRT-OsUBI-R | AAGGTGTTCAGTTCCAAGG |
| Gene cloning | OsKala3-F | ATGGGGAGGAAGCCGTGCTGCTCCAAGGAGGG |
|  | OsKala3-R | CTACTGGTGGTGGTGGTGGTGGTGGTGGTGGTGGTGGTC |
|  | OsKala4-F | ATGGCATCTGCTCCTCCAGTTCAG |
|  | OsKala4-R | TTACGGCGCCTTCCCCTGTCCAATTTCGGTT |
|  | OsTTG1-F | ATGGAGCAGCCCAAGCCGCCGTCGGT |
|  | OsTTG1-R | TCAGACCCTGAGAAGCTGGACCTTG |
| Subcellular localization | p326-OsKala3-F | CACGGGGGACTCTAGAATGGGGAGGAAGCCG |
|  | p326-OsKala3-R | CCATGGATCCTCTAGACTGGTGGTGGTGGTGGTGGTGGTGGTGGTGGTGGT |
|  | p326-OsKala4-F | CACGGGGGACTCTAGAATGGCATCTGCTCCTCCA |
|  | p326-OsKala4-R | CCATGGATCCTCTAGACGGCGCCTTCCCCTGTCC |
|  | p326-OsTTG1-F | CACGGGGGACTCTAGAATGGAGCAGCCCAAGCCG |
|  | p326-OsTTG1-R | CCATGGATCCTCTAGAGACCCTGAGAAGCTGGAC |
| Yeast two hybrid & Transactivation acticity | pGBKT7-OsKala3-F | CATGGAGGCCGAATTCATGGGGAGGAAGCCG |
|  | pGBKT7-OsKala3-N2-F | CATGGAGGCCGAATTCATCAAGAGGGGCAAC |
|  | pGBKT7-OsKala3-C-F | CATGGAGGCCGAATTCGCCATGCAACGGACC |
|  | pGBKT7-OsKala3-N1-R | GGATCCCCGGGAATTCCCCCGGCCGCAGGTA |
|  | pGBKT7-OsKala3-N2&N3-R | GGATCCCCGGGAATTCGACCCGCTTGCTGAG |
|  | pGBKT7-OsKala3-R | GGATCCCCGGGAATTCCTACTGGTGGTGGTGGTGGTGGTGGTGGTGGTGGTGGTC |
|  | pGADT7-OsKala4-F | GGAGGCCAGTGAATTCATGGCATCTGCTCCT |
|  | pGADT7-OsKala4-W-F | GGAGGCCAGTGAATTCTCTCCAGACGAAACA |
|  | pGADT7-OsKala4-C-F | GGAGGCCAGTGAATTCGTGGCAATAACGACG |
|  | pGADT7-OsKala4-M-R | CACCCGGGTGGAATTCAGGAGTAGAACTCGG |
|  | pGADT7-OsKala4-W&N-R | CACCCGGGTGGAATTCCTACCACGATCATCA |
|  | pGADT7-OsKala4-R | CACCCGGGTGGAATTCTTACGGCGCCTTCCC |
|  | pGBKT7-OsTTG1-F | CATGGAGGCCGAATTCATGGAGCAGCCCAAGCCG |
|  | pGBKT7-OsTTG1-R | GGATCCCCGGGAATTCGACCCTGAGAAGCTGGAC |
| PCR amplification of promoter & ORF region | OsKala3-pro-F | TCCGGTTTCTCTTCTGTTCG |
|  | OsKala3-1E-R | CGATCGAAGGCAAAGCTAGA |
|  | OsKala3-pro-F1 | ATGGGGAGGAAGCCGTGCTGCTCCAAGGAGGG |
|  | OsKala3-Full-R1 | GATGGATGCCGCTATCAGAGGAGCCATGG |
| Promoter activation | ProOsCHS-F | GCTGTCCCTAGCAAAAGCACCAGCA |
|  | ProOsCHS-R | CTCTCTCGACTAATTCACCAGGTCAC |
|  | ProOsCHI-F | CCATGGCACAATACACTTGCTTCCC |
|  | ProOsCHI-R | GGATACAGGATCGGGTGAGCAACTA |
|  | ProOsF3H-F | CTGGTATTTTGACGGGCTGTTGCGAC |
|  | ProOsF3H-R | CTCGATCGTTCGGCATGAAGCACGCG |
|  | ProOsF3'H-F | TTGATGCTCCTCCTCTAGTCGGCGACGT |
|  | ProOsF3'H-R | GACCGTATGATCCGCTCGCTCGTTGTACG |
|  | ProOsDFR-F | TACACTTCACGAACGACGCTGTCAT |
|  | ProOsDFR-R | GGCGTACCGTGCGTGATCGATCTGCTT |
|  | ProOsANS-F | CTGCGACCAACATGAAGAGACTATTGA |
|  | ProOsANS-R | GTCGCCCTCCCGAAGTAGGCTAGTA |
|  | pUC-ProOsCHS-fLUC-F | AGGCTCTAGAGGATCCACGGCGACACGGGAG |
|  | pUC-ProOsCHS-fLUC-R | TTGGCGTCTTCCATGGCTCTCTCGACTAATT |
|  | pUC-ProOsCHI-fLUC-F | AGGCTCTAGAGGATCCTTGCAGATGTTGGTTC |
|  | pUC-ProOsCHI-fLUC-R | TTGGCGTCTTCCATGGGGATACAGGATCGGG |
|  | pUC-ProOsF3H-fLUC-F | AGGCTCTAGAGGATCCTTGCACGGCACTTGA |
|  | pUC-ProOsF3H-fLUC-R | TTGGCGTCTTCCATGGCTCGATCGATCGACC |
|  | pUC-ProOsF3'H-fLUC-F | AGGCTCTAGAGGATCCTTGATGCTCCTCCTC |
|  | pUC-ProOsF3'H-fLUC-R | TTGGCGTCTTCCATGGGACCGTATGATCCGC |
|  | pUC-ProOsDFR-fLUC-F | AGGCTCTAGAGGATCCTACACTTCACGAACG |
|  | pUC-ProOsDFR-fLUC-R | TTGGCGTCTTCCATGGGGCGTACCGTGCGTG |
|  | pUC-ProOsANS-fLUC-F | AGGCTCTAGAGGATCCAGGACAACTGGTTGG |
|  | pUC-ProOsANS-fLUC-R | TTGGCGTCTTCCATGGGTCGCCCTCCCGAAG |
|  | ProOsKala3-1&2RU-F | TCCGGTTCTCTTCTGTTCG |
|  | ProOsKala3-1&2RU-R | CGATCGAAGGCAAAGCTAGA |
|  | pUC-ProOsKala3-1&2RU-fLUC-F | AGGCTCTAGAGGATCAGCGTGGTTTGATTTTTCA |
|  | pUC-ProOsKala3-1&2RU-fLUC-R | TTGGCGTCTTCCATGCCACGATTGCTCGATCGATCG |
|  | ProOsKala3-4RU-fLUC-F | AAGCTACTACGGATCCTATCGATGTCATAATATGTTAATT |
|  | ProOsKala3-8RU-fLUC-F | CACTCTGCAAATCAAAGCTACTACGGATCCGGG |
|  | ProOsKala3-4&8RU-fLUC-R | TCGATTTTCTGGATCAATGGCGGCAGCGTCTCT |
| Rice seed discrimination marker | CAPS-OsDFR-F | GAAGTAAGGAAGGAGGAGGAGCTTGCTTGACTCTGACAAGCTTGCTTGACTCTGACAA |
|  | CAPS-OsDFR-R | AAGGTGTTCAGTTCCAAGG |
|  | InDel-OsKala3-F | CAGCGTGGTTTGATTTTTCA |
|  | InDel-OsKala3-R | CGATCGAAGGCAAAGCTAGA |
|  | InDel-OsKala4-W&R-F | TCCTCTTCGTCGTTCTTGTCG |
|  | InDel-OsKala4-B-F | CCAATGGCAGAGACAGGTTCT |
|  | InDel-OsKala4-R | GAGTCTCTGTCCGGTTACGTC |
|  | InDel-OsRc-F | CAGGCACCACACAGAGAATG |
|  | InDel-OsRc-R | GGTTGGCACTGAAATCACCT |
